# Denosumab versus romosozumab for postmenopausal osteoporosis treatment

**DOI:** 10.1038/s41598-021-91248-6

**Published:** 2021-06-03

**Authors:** Tomonori Kobayakawa, Akiko Miyazaki, Makoto Saito, Takako Suzuki, Jun Takahashi, Yukio Nakamura

**Affiliations:** 1 Kobayakawa Orthopedic and Rheumatologic Clinic, 1969 Kunou, Fukuroi, Shizuoka 437-0061 Japan; 2 Department of Orthopaedic Surgery, Shinshu University School of Medicine, 3-1-1 Asahi, Matsumoto, Nagano 390-8621 Japan; 3 Department of Clinical Support Office, Tokyo Metropolitan Cancer and Infectious Diseases Center Komagome Hospital, 3-18-22 Honkagome, Bunkyou-ku, Tokyo 113-8677 Japan; 4 Department of Human Nutrition, Faculty of Human Nutrition, Tokyo Kasei Gakuin University, 22 Sanban-cho, Chiyoda-ku, Tokyo 102-8341 Japan

**Keywords:** Endocrinology, Medical research

## Abstract

Denosumab and romosozumab, a recently approved new drug, are effective and widely known molecular-targeted drugs for postmenopausal osteoporosis treatment. However, no studies have directly compared their therapeutic effects or safety in postmenopausal osteoporosis. This retrospective observational registry study compared the efficacy of 12-month denosumab or romosozumab treatment in postmenopausal osteoporosis patients. The primary outcome was the change in bone mineral density (BMD) at the lumbar spine. Secondary outcomes included BMD changes at the total hip and femoral neck, changes in bone turnover markers, and adverse events. Propensity score matching was employed to assemble patient groups with similar baseline characteristics. Sixty-nine patients each received either denosumab or romosozumab for 12 months. The mean 12-month percentage change from baseline in lumbar spine BMD was 7.2% in the denosumab group and 12.5% in the romosozumab group, indicating a significant difference between the groups. The percentage changes in BMD at both the total hip and femoral neck were also significantly higher at 12 months in the romosozumab group than in the denosumab group. In denosumab patients, bone formation and bone resorption markers were significantly decreased at 6 and 12 months from baseline. In the romosozumab group, the bone formation marker was significantly increased at 6 months and then returned to baseline, while the bone resorption marker was significantly decreased at both time points. Adverse events were few and predominantly minor in both groups, with no remarkable difference in the incidence of new vertebral fractures. Romosozumab showed a higher potential for improving BMD than denosumab in this clinical study of postmenopausal osteoporosis patient treatment.

## Introduction

At over 80 years, the average life expectancy in Japan is one of the highest in the world. However, healthy life expectancy remains in the early 70 s, indicating a period of approximately 10 years that may require some kind of medical care. One reason for this gap is a decrease in activities of daily living due to fragility fractures associated with osteoporosis^1–3^.

Approaches to osteoporosis treatment have become highly diversified, with clinicians now being able to offer tailor-made treatment options for each patient. As a new therapeutic goal, it is necessary to formulate osteoporosis treatments that elevate T-score to >  −2.5 within 5 years^4^, and so stronger therapeutic regimens may be required for patients with severely low bone mineral density (BMD).

In recent clinical practice, denosumab^5–7^ and romosozumab^8–10^ have become well-known molecular-targeted drugs with potent BMD-increasing effects. The drugs are considered especially important for osteoporosis management. However, no studies have directly compared the efficacy of denosumab and romosozumab in a clinical setting. The present study focused on comparisons of BMD improvement as an index of therapeutic efficacy since a greater increase in BMD would presumably lead to augmented prevention of fragility fractures^11^. Patients with a history of fragility fractures often exhibit a high rate of re-fracture occurrence within 1 year^12,13^. Therefore, it is considered clinically meaningful to investigate how denosumab and romosozumab work for improving BMD in a 1-year study period. Such data on postmenopausal osteoporosis may assist clinicians in making more appropriate treatment proposals to patients with osteoporosis.

## Methods

### Study population

From April 2015 to August 2020, this retrospective observational cohort study was conducted at our clinic and 4 affiliated institutions. The subjects were postmenopausal osteoporosis patients who were administered denosumab or romosozumab for 12 months.

Propensity score matching^14^ was performed for drug comparisons to reduce the differences in baseline characteristics between the groups. Propensity scores were estimated using a non-parsimonious multivariable logistic regression model. The variables considered for propensity score matching were age, body mass index (BMI), lumbar spine BMD, prevalent vertebral fracture, and prior non-vertebral fracture after 45 years of age. Denosumab (60 mg, s.c. once every 6 months) and romosozumab (210 mg, s.c. once every month) were used to treat patients diagnosed as having postmenopausal osteoporosis. Under the diagnosis of osteoporosis, subjects with one or more existing vertebral, total hip, or femoral neck fracture, or evidence of osteoporosis based on BMD T-score <  −2.5 at the lumbar spine, total hip, or femoral neck as measured by dual-energy X-ray absorptiometry (DXA), were recruited in this study^15^. Romosozumab administration is typically limited to patients with severe osteoporosis and a high risk of further fractures in Japan^16–20^. As exclusion criteria, male patients, secondary osteoporosis patients, especially those with any disease or medication that could influence bone turnover, patients with any cardiovascular events within the previous year, and patients with hypocalcemia were excluded. Patients with lower 25OHD values were offered an active vitamin D3 analogue, and if not inclined, were recommended to take commercially available vitamin D3 and calcium supplements. The study protocol of this investigation was reviewed by the ethics committee of Shinshu University School of Medicine and Kobayakawa Orthopedic and Rheumatologic Clinic. Written informed consent was obtained from all participants prior to enrollment. This study was conducted following the tenets outlined in the Declaration of Helsinki.

### Bone mineral density measurements

To evaluate the effects of 12-month osteoporosis therapy on BMD as the primary and secondary outcomes of interest, a Prodigy Fuga DXA device (GE Healthcare, Madison, WI, USA) was uniformly used at all participating institutions. The minimum significant change for this model was 2%^21^. Lumbar vertebra DXA measured the lumbar 2–4 levels and excluded any vertebral body with a T-score of 1.0 higher than the vertebral body with the lowest T-score. DXA readings were taken at baseline and at 6 and 12 months of treatment.

### Primary and secondary outcomes of interest

The primary outcome of interest was the percentage change from baseline in areal BMD by DXA at the lumbar spine during 12 months of treatment (mean values at 6 and 12 months). The secondary outcomes were the percentage changes in total hip and femoral neck BMD at 6 and 12 months as well as the percentage changes in the serum bone turnover markers procollagen type 1 N-terminal propeptide 1 (P1NP) and tartrate-resistant acid phosphatase isoform 5b (TRACP-5b). A previous report demonstrated that TRACP-5b levels were useful bone resorption markers that demonstrated higher clinical sensitivity and signal-to-noise ratio as compared with serum CTX levels^22^. P1NP and TRACP-5b were measured by the enzyme immunoassay and chemiluminescent enzyme immunoassay methods, respectively, at the time of treatment introduction (baseline) and at 6 and 12 months afterwards.

### Statistical analysis

Patient background parameters are expressed as the mean ± standard deviation. P1NP and TRACP-5b are expressed as the median. Percentage changes from baseline to the 6- and 12-month time points for BMD, P1NP, and TRACP-5b were assessed using the Wilcoxon signed-rank test. The Wilcoxon rank-sum test was employed to evaluate the differences between the groups with regards to the percentage changes from baseline for the primary and secondary outcomes. Differences between the study groups were determined by ANOVA or Fisher’s exact test. A two-tailed P-value of < 0.05 was considered statistically significant for all analyses. All statistical testing was conducted using R version 3.6.0 (R Core Team, 2019; http://www.R-project.org/).

## Results

### Study proportions

**Figure 1:**
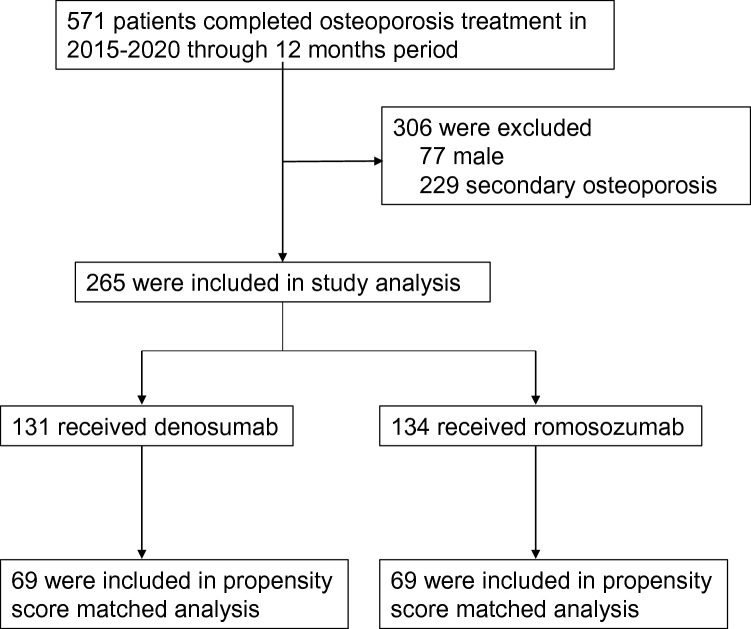
Subject flow diagram throughout the 12-month denosumab and romosozumab treatment period. Propensity score matching was employed to extract subjects.

Between April 2015 and August 2020, 571 osteoporosis patients received denosumab or romosozumab treatment for 12 months (Fig. 1). Seventy-seven male osteoporosis patients and 229 secondary osteoporosis patients were excluded, leaving 265 patients who met the inclusion criteria of this study. Of them, 131 received denosumab and 134 received romosozumab. Before propensity score matching, there were significant differences between the treatment groups for several baseline variables (Table 1), including a lower T-score and higher incidence of prior fragility fracture in the romosozumab group. These factors confirmed that romosozumab was used in patients with a higher risk of further fractures. After propensity score matching, 69 patients each had received denosumab or romosozumab. No remarkable differences in patient background were detected between the groups (Table 2). Mean ± standard deviation age was 74.2 ± 11.3 years in the denosumab group and 75.8 ± 9.70 years in the romosozumab group. Respective mean T-scores for the denosumab group and romosozumab group were − 2.50 ± 1.13 and − 2.62 ± 1.25 for the lumbar spine, − 2.55 ± 0.73 and − 2.57 ± 0.84 for the total hip, and − 3.12 ± 0.62 and − 3.12 ± 0.82 for the femoral neck. Twenty-six (37.7%) subjects had a prevalent vertebral fracture in the denosumab group, as compared with 25 (36.2%) subjects in the romosozumab group. Twelve (17.4%) and 13 (18.8%) subjects had a history of prior non-vertebral fracture in the denosumab group and romosozumab group, respectively. Twenty-seven (39.1%) denosumab patients and 20 (29.0%) romosozumab patients had some kind of osteoporosis treatment history and had been switched to either denosumab or romosozumab without a set washout period. The number of subjects who were treatment naïve was 42 (60.9%) in the denosumab group and 49 (71.0%) in the romosozumab group.

**Table 1:** Demographic and clinical characteristics of subjects at baseline before extraction by propensity score matching.

	Denosumab (N = 134)	Romosozumab (N = 131)	P value
Age (years, mean ± SD)	73.1 ± 12.3	76.3 ± 8.7	0.017
BMI (kg/m^2^)	21.2 ± 3.2	21.6 ± 3.3	0.561
**T-score**			
Lumbar spine	− 2.11 ± 1.22	− 2.89 ± 1.11	< 0.001
Total hip	− 2.48 ± 0.69	− 2.62 ± 0.81	0.247
Femoral neck	− 2.94 ± 0.64	− 3.19 ± 0.80	0.003
Prior vertebral fracture, n (%)	42 (31.3)	57 (43.5)	0.043
Prior non-vertebral fracture, n (%)	17 (12.7)	40 (30.5)	0.001
**History of prior treatment, n (%)**			
Naïve	90 (67.2)	84 (64.1)	0.608
Switch	44 (32.8)	47 (35.9)	
Concomitant use of active vitamin D, n (%)	22 (55.0)	62 (47.7)	0.472
PINP (µg/L, median)	56.8 [39.0, 75.1]	67.3 [41.3, 95.9]	0.062
TRACP-5b (mU/dL, median)	458.5 [365.0, 642.3]	522.0 [341.5, 665.0]	0.491
Serum albumin (g/dL)	4.1 ± 0.3	4.2 ± 0.3	0.031
Serum-corrected Ca (mg/dL)	9.4 ± 0.5	9.3 ± 0.4	0.006
eGFR (mL/min/1.73 m^2^)	69.8 ± 21.3	67.6 ± 19.7	0.491
25OHD (ng/mL)	15.6 ± 7.0	16.3 ± 6.3	0.226

Data are expressed as the mean ± standard deviation (SD) or the number (%) of all patients who completed 12 months of denosumab or romosozumab treatment. P1NP and TRACP-5b are expressed as median values.

*BMI* body mass index, *P1NP* procollagen type 1 N-terminal propeptide, *TRACP-5b* tartrate-resistant acid phosphatase isoform 5b, *eGFR* estimated glomerular filtration rate, *25OHD* 25-hydroxyvitamin D; Differences between the groups were determined by ANOVA or Fisher’s exact test.

**Table 2:** Demographic and clinical characteristics of subjects at baseline after extraction by propensity score matching.

	Denosumab (n = 69)	Romosozumab (n = 69)	P value
Age (years, mean ± SD)	74.20 ± 11.32	75.83 ± 9.70	0.367
BMI (kg/m^2^)	21.15 ± 3.39	22.09 ± 3.24	0.192
**T-score**			
Lumbar spine	− 2.50 ± 1.13	− 2.62 ± 1.25	0.322
Total hip	− 2.55 ± 0.73	− 2.57 ± 0.84	0.930
Femoral neck	− 3.12 ± 0.62	− 3.12 ± 0.82	0.870
Prior vertebral fracture, n (%)	26 (37.7)	25 (36.2)	1.000
Prior non-vertebral fracture, n (%)	12 (17.4)	13 (18.8)	1.000
**History of prior treatment, n (%)**			
Naïve	42 (60.9)	49 (70.1)	0.281
Switch	27 (39.1)	20 (29.0)	
Concomitant use of active vitamin D, n (%)	12 (17.4)	35 (50.7)	0.612
PINP (µg/L, IQR)	56.8 [35.9, 84.3]	68.6 [41.3, 99.8]	0.086
TRACP-5b (mU/dL, IQR)	454.0 [350.5, 621.5]	545.0 [353.0, 690.0]	0.296
Serum albumin (g/dL)	4.12 ± 0.29	4.17 ± 0.34	0.104
Serum-corrected Ca (mg/dL)	9.35 ± 0.47	9.34 ± 0.37	0.761
eGFR (mL/min/1.73 m^2^)	70.36 ± 21.43	68.78 ± 21.9	0.472
25OHD (ng/mL)	15.00 ± 6.32	16.35 ± 6.17	0.241

Data are expressed as the mean ± standard deviation (SD) or the number (%) of subjects. P1NP and TRACP-5b are expressed as median values.

*BMI* body mass index, *P1NP* procollagen type 1 N-terminal propeptide, *TRACP-5b* tartrate-resistant acid phosphatase isoform 5b, *eGFR* estimated glomerular filtration rate, *25OHD* 25-hydroxyvitamin D; Differences between the groups were determined by ANOVA or Fisher’s exact test.

### Primary outcome

**Figure 2:**
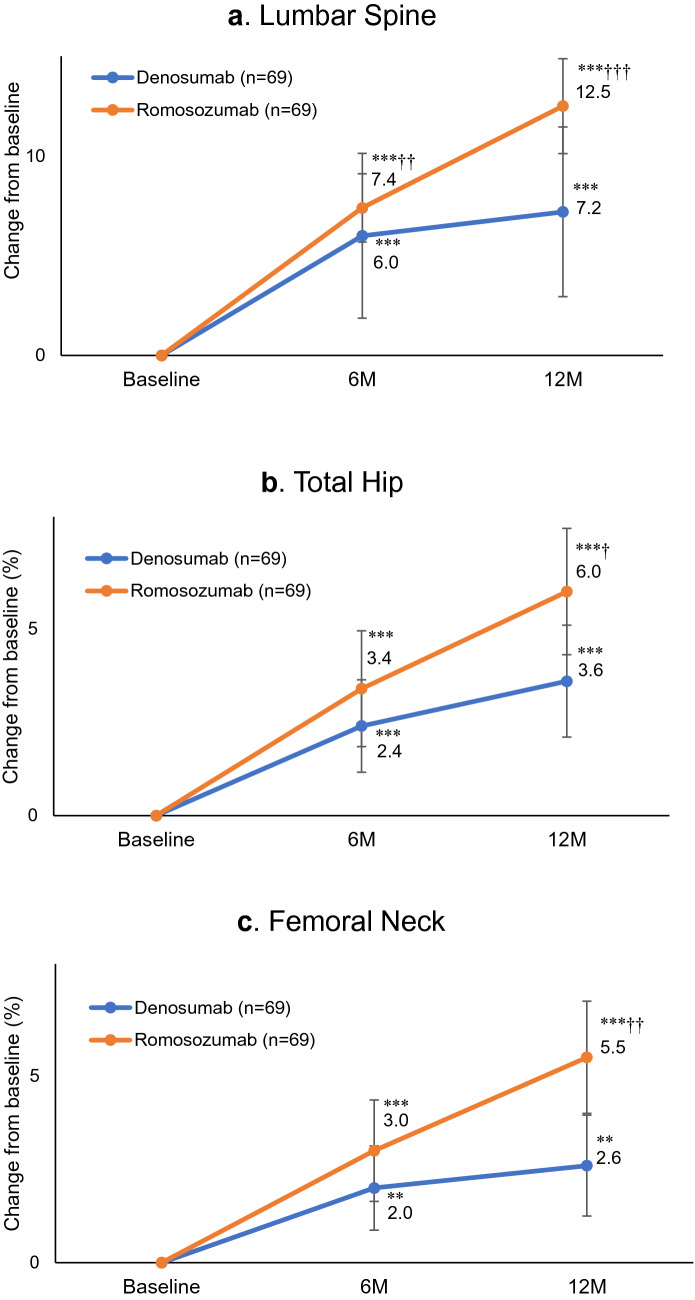
Mean percentage changes from baseline to 6 and 12 months (M) in bone mineral density (BMD) at the (**a**) lumbar spine, (**b**) total hip, and (**c**) femoral neck. Bars indicate the mean ± 95% confidence interval.  **P < 0.01 and ***P < 0.001 versus baseline (Wilcoxon's signed-rank test). ^†^P < 0.05, ^††^P < 0.01, and ^†††^P < 0.001 versus denosumab (Wilcoxon's rank-sum test).

Sixty-nine patients each in the denosumab group and romosozumab group were included in the primary outcome analysis. The respective percentage changes from baseline (mean ± 95%CI) in areal BMD tested by DXA at the lumbar spine at 6 and 12 months were 6.0% ± 4.1 (P < 0.001 versus baseline) and 7.2% ± 4.3 (P < 0.001 versus baseline) in the denosumab group and 7.4% ± 1.7 (P < 0.001 versus baseline) and 12.5% ± 2.4 (P < 0.001 versus baseline) in the romosozumab group (Fig. 2a). The percentage change in lumbar spine BMD was significantly higher in the romosozumab group than in the denosumab group at 6 (P < 0.01) and 12 months (P < 0.001).

### Secondary outcomes

The respective percentage changes in total hip BMD from baseline at 6 and 12 months were 2.4% and 3.6% in the denosumab group and 3.4% and 6.0% in the romosozumab group (Fig. 2b). Similar results of 2.0% and 2.6% in the denosumab group and 3.0% and 5.5% in the romosozumab group were observed for BMD at the femoral neck (Fig. 2c). All increases were significant (P < 0.01) versus baseline values for both drugs. There was no remarkable difference in percent increases between the denosumab group and romosozumab group at 6 months (total hip: P = 0.394, femoral neck: P = 0.331), although significant differences were noted at 12 months (total hip: P < 0.05, femoral neck: P < 0.01). Those data supported a possible advantage of romosozumab for elevating bone density over denosumab.

**Figure 3:**
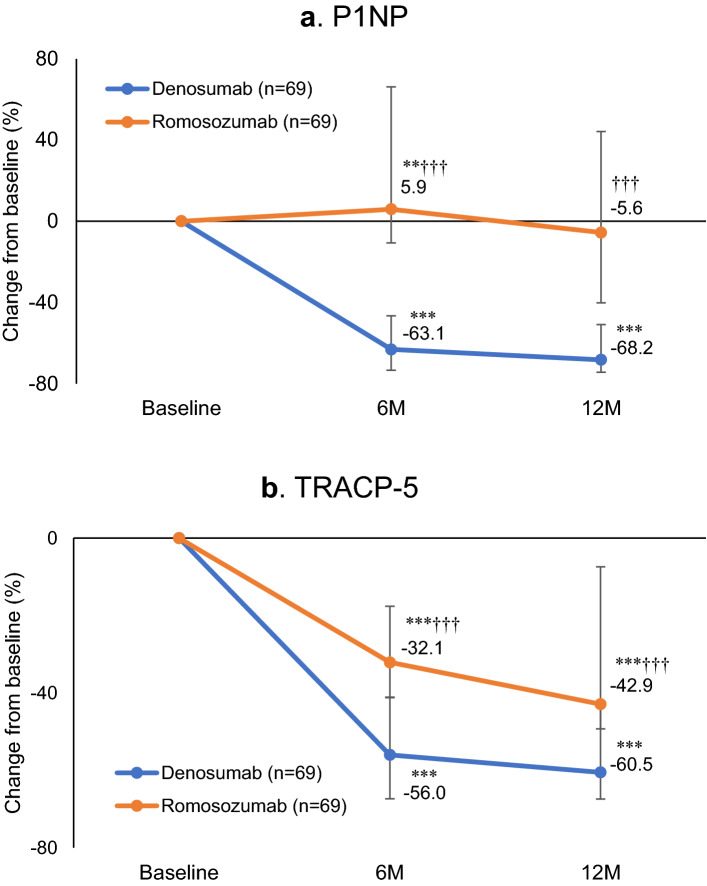
Median percentage changes from baseline to 6 and 12 months in (**a**) serum procollagen type 1 N-terminal propeptide (P1NP) level and (**b**) serum tartrate-resistant acid phosphatase isoform 5b (TRACP-5b). Bars indicate the median ± first or third quartile. **P < 0.01 and ***P < 0.001 versus baseline (Wilcoxon's signed-rank test). ^†††^P < 0.001 versus denosumab (Wilcoxon's rank-sum test).

Next, as the other important factors for osteoporosis treatment, the changes in major serum bone turnover markers, P1NP and TRACP by the action of these treatment drugs were focused. Serum P1NP level was significantly decreased at 6 months (− 63.1%; P < 0.001) and 12 months (− 68.2%; P < 0.001) compared with baseline in the denosumab group. In the romosozumab group, P1NP was significantly higher at 6 months (5.9%; P < 0.01), and then normalized at 12 months (− 5.6%; P = 0.705) (Fig. 3a). There were significant differences between the groups at 6 months (P < 0.001) and 12 months (P < 0.001). Serum TRACP-5b level in the denosumab group was significantly decreased at 6 months (− 56.0%; P < 0.001) and 12 months (− 60.5%; P < 0.001) versus baseline values (Fig. 3b). The romosozumab group displayed a similar trend at 6 months (− 32.1%; P < 0.001) and 12 months (− 42.9%; P < 0.001). A significant difference was observed between the groups both time points (both P < 0.001).

### Adverse events and new fractures

The adverse events recorded during the 12 months of treatment are listed in Table 3. Although injection site reactions occurred more frequently in the romosozumab group, they did not lead to drug discontinuation. Injection site reactions often occurred at the time of first administration. The presence of new vertebral fractures during the 12 months of treatment was evaluated by regular X-ray photographs. Two patients in each group (both 2.9%) suffered a new fracture, an incidence that was statistically comparable.

**Table 3:** Adverse events and new bone fractures during treatment.

	Denosumab (N = 69)	Romosozumab (N = 69)
All adverse events	6 (8.7)	25 (36.2)
**Serious adverse events**		
Breast cancer	0	1 (1.4)
Injection site reaction*		
Pain	0	10 (14.5)
Swelling	0	4 (5.8)
Redness	0	1 (1.4)
Itching	0	2 (2.9)
**Other events of interest**		
Anacatesthesia	0	1 (1.4)
Blindness	1 (1.4)	0
Numbness in limbs	1 (1.4)	0
Diarrhea	1 (1.4)	0
Blood pressure elevation	0	1 (1.4)
Fatigue	0	1 (1.4)
**New fractures during the therapy**		
Thoracic or lumbar spine	2 (2.9)	2 (2.9)
Proximal tibial fracture	0	1 (1.4)
Rib fracture	1 (1.4)	0
Distal fibular fracture	0	1 (1.4)

Data are expressed as the number of subjects (%).

*Injection site reactions included adverse events on the skin at the injection site lasting 2 days or longer.

## Discussion

Using a propensity score-matching cohort design, the present study found that the increasing rates of lumbar spine, total hip, and femoral neck BMD were significantly higher with romosozumab than with denosumab after a treatment period of 12 months, with few serious adverse effects for either drug.

Denosumab is a fully human monoclonal antibody to the receptor activator of nuclear factor-kappa B ligand (RANKL) that blocks its binding to RANK, thus inhibiting the development and activity of osteoclasts, decreasing bone resorption, and increasing bone density^23^. On the other hand, romosozumab is a bone-forming agent that inhibits sclerostin to promote bone formation and suppress bone resorption through a so-called “dual-effect”^24,25^. These molecular-targeted drugs are prominent in the field of osteoporosis treatment.

Three factors are involved in the increase of BMD: (1) initial closure of the bone remodeling space, (2) a subsequent increase in mineralization, and (3) the steady contribution of modeling-based bone formation^26,27^. Especially in bone remodeling, the transition of bone metabolism markers affects the size of the anabolic window due to the difference between the levels of bone formation markers and bone resorption markers^28^. Denosumab strongly suppresses bone resorption, which in turn inhibits bone formation as well. Bone remodeling is considered to proceed under these conditions. In contrast, romosozumab promotes bone formation and suppresses bone resorption, resulting in a larger anabolic window and presumably a greater effect on increasing bone density. In this study, denosumab decreased both the bone formation marker and the bone resorption marker, while the bone formation marker did not decrease throughout 12 months and only the bone resorption marker was decreased for romosozumab. Accordingly, we considered that a larger anabolic window was created. Romosozumab also had a greater effect on bone modeling than on bone remodeling in a recent report^29^. Taken together, the considerable effects of romosozumab on bone remodeling and modeling appear more effective to increase bone density levels. Both our primary and secondary clinical results support this theory.

As a notable point, the low levels of vitamin D in the cohort are not unique circumstances in Japan. In the real-world setting, approximately 90% of Japanese patients suffer from a vitamin D deficiency or insufficiency as a complication of osteoporosis^30^. We routinely advise the intake of active vitamin D or calcium preparations for patients with lower 25OHD; however, some patients find it undesirable to take additional medicine, and ultimately reject the prescription. For those individuals, we suggest other strategies, such as the intake of supplements and insolation, and provide suitable daily life guidance as well as further medical treatment. In addition, romosozumab is usually reserved for patients with severe osteoporosis in actual clinical practice, and we believe that immediate intervention is required without losing time waiting for 25OHD elevation to a sufficient level to prevent further fractures.

As limitations of this study, the following factors require further consideration: (1) there was no discussion on treatment-naïve vs. switch (non-naïve) patients because we focused on the standardization of patient background characteristics by propensity score matching, (2) as the observation period of this study was short at 1 year, longer follow-up for adverse events and new fractures is needed, and (3) the data on adverse events during the 12 months of treatment were obtained from the clinical records of patients, which was a retrospective process.

In conclusion, this investigation used propensity score matching to directly compare the clinical effects of denosumab and romosozumab in patients with postmenopausal osteoporosis. In terms of BMD of the lumbar spine, total hip, and femoral neck, the 12-month gains in the romosozumab group were all significantly higher than those in the denosumab group, indicating a potential therapeutic advantage that warrants further validation.
